# Efficient Removal of Hg(II) from Water under Mildly Acidic Conditions with Hierarchical SiO_2_ Monoliths Functionalized with –SH Groups

**DOI:** 10.3390/ma15041580

**Published:** 2022-02-20

**Authors:** Ireri Segura Gutiérrez, Verónica Hernández Morales, Eric Mauricio Rivera Muñoz, Rufino Nava Mendoza, Ludwig Lagarde Soto, Carmen Leticia Peza Ledesma, Doris Solís Casados, Barbara Pawelec

**Affiliations:** 1 División de Investigación y Posgrado, Facultad de Ingeniería, Universidad Autónoma de Querétaro, Centro Universitario, Cerro de las Campanas, Santiago de Querétaro 76000, Mexico; irerisegura@gmail.com (I.S.G.); ludwig.lagarde.soto@uaq.mx (L.L.S.); 2 Centro de Física Aplicada y Tecnología Avanzada, Universidad Nacional Autónoma de México, Campus Juriquilla, Santiago de Querétaro 76230, Mexico; vero_hm@hotmail.com (V.H.M.); emrivera@fata.unam.mx (E.M.R.M.); carmenpez@gmail.com (C.L.P.L.); 3 Centro Conjunto de Investigación en Química Sustentable UAEM-UNAM, Carretera Toluca-Atlacomulco Km 14.5, Unidad San Cayetano, Toluca 50200, Mexico; solis_casados@yahoo.com.mx; 4 Instituto de Catálisis y Petroleoquímica, CSIC, Cantoblanco, 28049 Madrid, Spain

**Keywords:** monoliths, adsorption, thiol groups, mercury removal

## Abstract

In this work, novel adsorbents based on 3D hierarchical silica monoliths functionalized with thiol groups were used for the removal of Hg(II) ions from an acidic aqueous solution (pH 3.5). Silica monoliths were synthesized by using two different pluronic triblock polymers (P123 and F127) to study the effect of porous structure on their sorption capacity. Before and after functionalization by grafting with 3-mercaptopropyltrimethoxysilane (MPTMS), the monoliths were characterized by several techniques, and their Hg(II) removal potential was evaluated in batch experiments at 28 °C and pH 3.5, using different initial concentrations of Hg(II) ions in water (200–500 mg L^−1^). The thiol groups of the monoliths calcined at 550 °C showed thermal stability up to 300 °C (from TG/DTG). The functionalized monolith synthesized with P123 polymer and polyethylene glycol showed favorable hierarchical macro-mesopores for Hg(II) adsorption. M(P123)–SH exhibited 97% removal of Hg(II) at concentration 200 mg L^−1^. Its maximum adsorption capacity (12.2 mmol g^−1^) was two times higher than that of M(F127)–SH, demonstrating that the 3D hierarchical macro-mesoporosity allowing accessibility of Hg(II) to thiol groups favors the physical and chemical adsorption of Hg(II) under slightly acidic conditions.

## 1. Introduction

The removal of toxic heavy metals from wastewater by adsorption technology is of great importance to public health. Among different heavy metals, mercury is one of most difficult to remove, and still there exists no rapid and effective technology for its elimination from environment [1]. In this regard, attempts were made to use low-cost natural lignocellulosic adsorbents, but these materials demonstrated low mechanical resistance to abrasive forces, low loading capacities and relatively weak interactions with heavy metal cations [1,2,3]. In addition, most of those materials exhibit very low porosity and surface area, which would largely restrict incorporation of the functional groups, leading to the reduction of the heavy-metal adsorption capacity of cellulose based adsorbents [4]. In this sense, the use as synthetic adsorbents of mesoporous silica materials with high porosity and surface area allows a faster transport of heavy metals from the external environment to the adsorption sites and offers a larger number of adsorption sites [5,6,7,8,9,10,11,12,13,14,15,16,17,18,19].

Recent advances in the field of sol–gel chemistry allows researchers to achieve macroscopic shape control in porous oxides such as those prepared as films, fibers and monoliths [20,21,22,23,24], thus increasingly opening up the range of applications. In this respect, materials with small and large pores arranged in a hierarchical structure are desirable materials. The pristine siliceous materials are not able to tramp Hg(II) ions. Therefore, surface functionalization of hierarchical siliceous materials can improve their adsorption capacity in these applications [25,26]. Among different chelating agents, the excellent results for Hg(II) removal was reported for silica materials functionalized with chelating mercapto groups [9,12,13,14,15,16,17,18,19,20,21,22,23,24,25,26,27,28,29,30,31,32,33,34,35,36,37,38,39]. For example, an effective mercury removal was archived by using the metal–organic framework adsorbent (MOF–808–SH) fabricated by grafting thioglycollic acid on MOF-808 [1]. The high adsorption capacity of this synthesized adsorbent was explained as being due to its large surface area, well-developed porous structures and high –SH loading [1]. Major advances in the synthesis of porous organic materials and their potential as adsorbents for the removal of Hg(II) from aqueous solution were recently reviewed by Modak et al. [17] and Da’na [18].

The preparation of mesoporous silica monoliths by micellar templating presents several advantages [25,26,27]. One of them is that, under specific synthesis conditions, surfactant assemblies can be crystallographically organized, thus allowing the pore orientation to be controlled [39]. Smarsly et al. [40] demonstrated that silica membranes prepared by using polystyrene-block-poly(ethylene oxide) (PS–b–PEO) as a templating agent resulted mainly in membranes with larger mesopores interconnected with micropores. Boissiere et al. [41] prepared silica membranes by using a polyethylene oxide (PEO)-based nonionic surfactant, resulting in 2.5 nm mesopores according to shear permeation experiments with PEO polymer solutions. Nakanishi [42] and Minakuchi et al. [43] used a wide variety of water-soluble polymers, such as poly(ethylene oxide) (PEO), to control the phase-separation/gelling kinetics in the preparation of monolithic silica of virtually any shape exhibiting both interconnected macropores and textural mesoporosity. Since the phase-separation period relative to the sol–gel transition determines the size of the macropores, the macropore diameter can be easily controlled by adjusting the polymer concentration.

It is well-known that pore size and pore surface chemistry are the main factors influencing the adsorbent capacity for heavy metal removal. For example, high adsorption capacity for heavy metal removal from wastewater presents silica aerogels that have a large surface area due to their extremely porous structure composed of small pores (micro- and mesopores) [23]. However, this situation can be expected to be different if the adsorbent is functionalized with long-chain chelating agents that can block the access of the adsorbate to the internal porous structure of the adsorbent. In this case, the presence of macropores allows the introduction of the functional groups into the porous structure of the adsorbent. Macroporous materials formed by silica nanoparticle aggregates exhibit textural mesoporosity, high specific surface area and pore sizes in the range of 10–20 nm. Mesopore size can be controlled almost independently of macropore size by post-synthesis treatment in an ammonia solution [43,44]. Since silica solubility depends on both pH and temperature, an increase in either will enhance Ostwald ripening of the silica matrix, leading to an increase in mesoporous diameter. In this sense, the recent study by Xia et al. demonstrated that a high surface area and abundant macro/meso-porosities of the adsorbate are necessary conditions for the efficient Hg ions trapping [4]. 

From the literature review [17,18], we observed that most of the synthesized sorbents are in powder form, which is not so cost-effective in terms of expensive filtration and separation equipment. Another challenge is how to avoid structural collapse of the adsorbent during the adsorption or regeneration steps [18]. All of these problems could be solved by using monolithic silica adsorbents, as is demonstrated in this work by synthesizing new inorganic/organic silica adsorbents grafted with propylthiol groups. The ordered silica materials, such as SBA–16, demonstrated good regeneration behavior [32], and it is expected that its compact monolithic structure can prevent the structural collapse of the adsorbent during the adsorption or regeneration stages. To our knowledge, the use of adsorbents with this design has not been explored. The idea was the same as that of composites based on conductive polymers or carbon nanomaterials designed for the detection of heavy metal ions [45,46]

In this work, we prepared monolithic silica adsorbents exhibiting multimodal porosity, using a tri-block EO–PO–EO copolymer surfactant similar to that used for preparation of SBA–15 (Pluronic P123) and SBA–16 (Pluronic F127) type materials, one with hexagonal pore arrangement and the other with cubic pore arrangement. The monolithic adsorbents offer advanced textural properties for easy access of Hg(II) ions to the –SH groups. In this regard, short-channel SBA–15 functionalized with thiol groups were found to be more efficient for Hg(II) removal than long-channel SBA–15–SH [32]. The silica monoliths were prepared by the sol–gel method, using polyethylene glycol (PEG, 20,000) as a phase-separation agent and to avoid particle aggregation. Hierarchical monoliths functionalized with –SH groups were used as adsorbents for the removal of mercury from an aqueous solution. Since there are studies showing that heavy metal removal decreases under acidic conditions (pH < 4) and that the optimum pH of the aqueous mercury solution could be archived at pH = 4–6 [47], the objective was to study the effect of adsorbent porosity on its efficiency for mercury removal at pH 3.5.

## 2. Experimental

### 2.1. Preparation of Hierarchical Silica Monoliths

Hierarchical silica monolithic material was synthesized by using tetraethyl orthosilicate (TEOS, 98%, Sigma-Aldrich, St. Louis, MO, USA) as the silica source, while polyethylene glycol (PEG) with an average 20,000 g/mol molecular weight (J.T. Baker, Center Valley, PA, USA) was utilized as the phase-separation agent. Non-ionic pluronic triblock pluronic co-polymers (BASF, EO_20_–PO_70_–EO_20_, P123, Florham Park, NJ, USA) and (BASF, EO_106_–PO_70_–EO_106_, F127, Florham Park, NJ, USA) were used as structure-directing agents to produce mesopores in the range of 3–8 nm.

Silica monoliths were prepared by adding P123 or F127 to a mixture of PEG dissolved in 1 M aqueous nitric acid solution (65%, J.T. Baker, Center Valley, PA, USA), which was used as a catalyst for hydrolysis and condensation of pluronic triblock polymers and TEOS. The mixture was homogenized by stirring at room temperature. After this, the required amount of silica precursor (TEOS/P123 molar ratio of 59; TEOS/F127 molar ratio of 166) was added dropwise to the solution under vigorous stirring at 40 °C for 10 min and kept under slow stirring for 60 min at the same temperature. The resulting mixture was transferred to polypropylene vessels and kept at room temperature for 72 h, and then aged for 24 h at 60 °C. Preservation of the monoliths was carried out in a solution of 1M NH_4_OH (ammonia 30%, J. T. Baker, Center Valley, PA, USA) for 24 h at 35 °C. These elements were then acidified with a 0.1 M HNO_3_ solution and washed with ethanol (25 wt.%). Finally, the silica monoliths were dried at 60 °C for 7 h and then at 110 °C for 24 h. The polymer was removed by calcination in air at 550 °C for 5 h. Depending on the polymer used, the adsorbents will be coded hereafter as M(P123) and M(F127), where M means “monoliths”.

### 2.2. Preparation of Thiol-Functionalized Adsorbents

Hierarchical mercapto-containing monoliths were synthesized using 3-mercaptopropyl-trimethoxysilane (MPTMS: SH(CH_2_)_3_Si(OMe)_3_; 95%) in absolute ethanol (Sigma-Aldrich Co., St Louis, MO, USA) as a source of –SH groups. Each thiol-functionalized hybrid material was prepared with the solutions of equal concentration of MPTMS to obtain samples with the molar composition of TEOS-to-MPTMS 1:0.3 and the same content of –SH groups in both monoliths. The concentration of MPTMS corresponds to the theoretically necessary concentration for efficient adsorption of Hg (II) [48]. The hierarchical monolith (1 g) was immersed in MPTMS–ethanol solution at room temperature in an inert atmosphere for 30 min. Deionized water was then slowly added, and the hierarchical monolith remained in solution for 24 h. Water was incorporated to carry out the hydrolysis of the alkoxy groups of MPTMS. The amount of water added was twice the amount needed to completely hydrolyze the MPTMS load. Finally, the solid was dried at room temperature, at 110 °C, for 24 h. Monoliths (Ms) functionalized with thiol groups are coded hereafter as M(P123)–SH and M(F127)–SH.

### 2.3. Mercury Batch Adsorption Experiments

The adsorption capacity of the synthesized hierarchical silica monoliths was evaluated by mercury batch adsorption tests, following the method described by Hernandez-Morales et al. [48]. The mercury stock solution was prepared by dissolving Hg(NO_3_)_2_·H_2_O (J.T. Baker, Center Valley, PA, USA) in deionized water, while aqueous solutions with Hg(II) concentration of 200, 300, 400 and 500 mg L^−1^ were prepared by diluting this stock solution with deionized water. All adsorption experiments were carried out at 28 °C, pH 3.5 and contact time of 1 h. This contact time was selected because, prior to the adsorption experiments, the effect of contact time on the adsorption capacity of the functionalized monoliths was investigated for adsorption times within the range of 0–120 min. The results showed that the adsorption process of Hg(II) reached a rapid equilibrium at about 40 min. In a typical run, 0.1 g of sorbent was dispersed in 100 mL of aqueous mercury solution. After the contact time of 1 h, the solid was separated by filtration. The pH was kept constant around 3.5 by adding drops of concentrated HCl if necessary. Mercury concentration before and after adsorption experiments was determined by atomic adsorption spectroscopy, using a hydride generator (AA–H) (Perkin Elmer, Waltham, MA, USA) and a Perkin Elmer Optima 3300 DV spectrometer (Perin Elmer, Waltham, MA, USA) calibrated with stock solution. The emission lines used were in accordance with the EPA standard method for metals analysis [49]. Considering the extensive information reported in the literature on the regeneration of thiol-functionalized ordered mesoporous silica [3], the good regeneration of monoliths could be expected.

### 2.4. Characterization Methods

To examine the textural properties and surface functionality, the prepared monoliths were characterized by using various techniques: Scanning electron microscopy (SEM) (Hitachi Ltd., Tokyo, Japan), Fourier-transform infrared spectroscopy (FTIR) (Bruker, Billerica, MA, USA) and thermogravimetric analysis (TGA/DTA; TA Instruments, SDT Q 600 model, New Castle, DE, USA). The specific surface areas of the monoliths were calculated by applying the BET (Brunauer–Emmett–Teller) equation to nitrogen adsorption desorption isotherms [50] obtained at −196 °C within the relative pressures 0.03 < *P*/*P*_0_ < 0.30 (Acusorb Quantachrome Instruments equipment, Graz, Austria). Prior to the analysis, the sample was degassed at 200 °C, under vacuum, for 18 h to assure a dry, clean surface that is free of weakly adsorbed species. The pore size distribution was analyzed by using the Barrett–Joyner–Halenda (BJH) model [51]. The total pore volume (*V*_total_) was estimated from the amount of nitrogen adsorbed at a 0.99 relative pressure. The surface morphology and chemical composition were evaluated by scanning electron microscopy (SEM images) performed on a HITACHI SU8200 microscope (Hitachi Ltd., Tokyo, Japan) at electron accelerating voltage of 1 kV and by employing the working distance of 1.7 mm. Powder samples were placed in the sample holder with silver adhesive to improve electrical contact. Fourier-transform IR (FTIR) spectra of P123 and F127 monoliths and SH–functionalized adsorbents were recorded in the range 400–1800 cm^−1^ on a Bruker Vector 3.3 (Billerica, MA, USA) spectrophotometer, using the KBr wafer technique. The influence of temperature on the stability of the SH groups was determined by applying thermogravimetric analysis on a Model TGA 2950 high-resolution thermogravimetric analyzer V5.4a, Columbus, OH, USA) by increasing temperature from 25 to 600 °C (heating rate of 5 °C/min) under nitrogen flow. The UV–Vis diffuse reflectance spectra of the adsorbents after Hg (II) adsorption were recorded at RT on an Ocean Optics, Inc. spectrometer First in Photonics (Mini-DT 2, Waltham, MA, USA). The elemental composition of the adsorbents before and after mercury adsorption was analyzed by X-ray photoelectron spectroscopy (XPS) on the Jeol JPS 9200 XPS (Akishima, Tokyo, Japan) apparatus, using an Mg Ka (*h*υ = 1253.6 eV; 1 eV = 1.603 × 10^−19^ J) X-ray radiation source and a C 1s peak centered at 284.8 eV for correction.

## 3. Result and Discussion 

### 3.1. Sorbent Characterization

#### 3.1.1. Textural Properties

**Figure 1:**
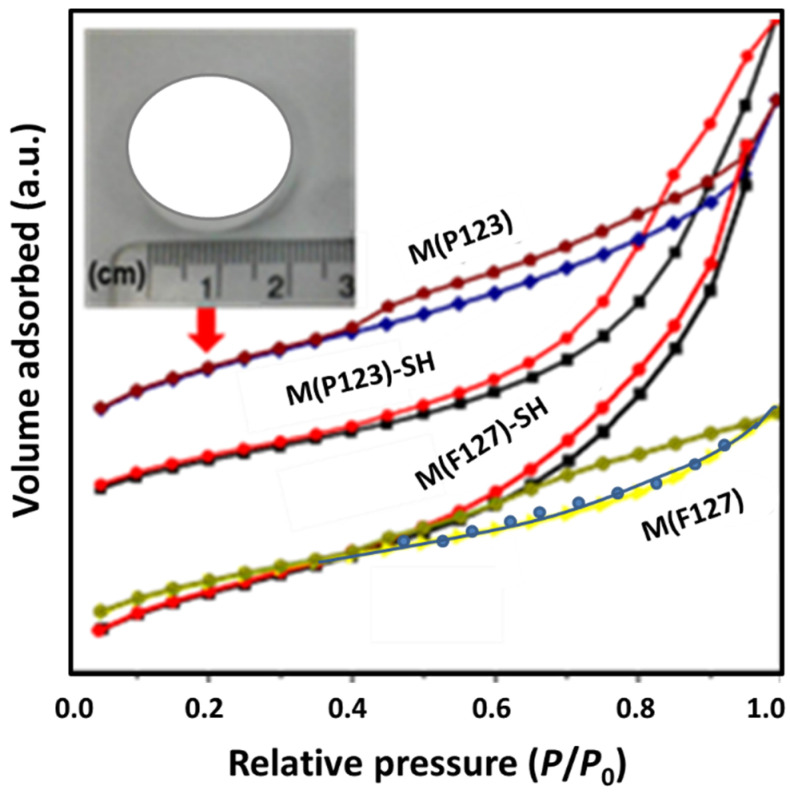
N_2_ adsorption–desorption isotherms of the hierarchical monoliths before and after functionalization with mercapto groups. The image of M(P123) tablet with 2.5 cm diameter is shown in the inlet of this figure.

For both pristine and thiol functionalized monolithic adsorbents, the low angle X-ray diffraction (figure not shown here) confirmed the unique presence of 2*θ* (100) plane, indicating structures with poorer order. The diffractograms were similar to those reported for highly effective SBA–15–SH adsorbents [30]. The textural properties of all sorbents were evaluated by nitrogen adsorption–desorption isotherms at −196 °C (Figure 1). According to the IUPAC classification [52], all samples showed type III isotherms. The isotherms showed a well-defined region in the relative pressure range (0.4 < *P*/*P*_0_ < 0.9), where *P* is the equilibrium adsorption pressure, and *P*_0_ is the saturation pressure representing spontaneous filling of the pores due to capillary condensation. The isotherms of the pure samples showed well-defined steps of the desorption branch, indicating the existence of an open pore structure, whereas the functionalized samples showed undefined steps of the desorption branch, suggesting the plugging of the pores by the ligand. As a consequence of the limited access of N_2_ molecules to the micropores, the calculation of the mean pore diameter does not include the diameter of the micropores being the mean pore diameter larger than that of the pristine monoliths. The shape of the hysteresis loop suggests hierarchical porosity of the materials with an interconnected pore structure [53]. As an example, the image of tablet M(P123) with 2.5 cm diameter is shown in the inlet of Figure 1. As can be seen, due to the synthesis procedure used for the formation of a silica gel, the monolith maintains the size and shape of the container used.

**Figure 2:**
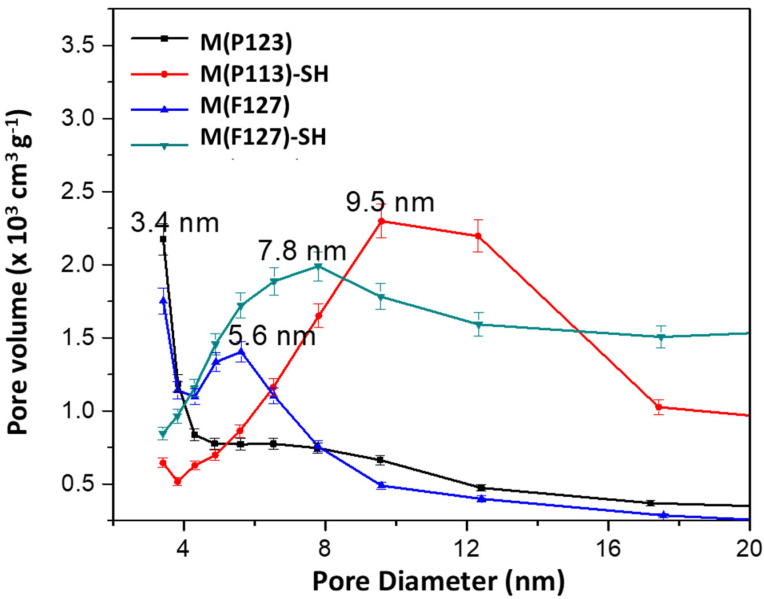
Pore size distributions of the hierarchical silica monoliths before and after functionalization with –SH groups.

The specific surface area (*S*_BET_), pore size (*d*) and total pore volume (*V*) are shown in Table 1. *S*_BET_ values were calculated from nitrogen sorption studies by applying the BET equation for specific surface area [50] and the BJH formula for pore size distribution [51]. It is noteworthy that the content of –SH groups was the same in both monoliths. Although Pluronic F127 has a higher number of EO chains than pluronic P123, the total pore volume and *S*_BET_ of M(F127) is only slightly lower than that of M(P123), and their average pore diameter is similar. This situation is changed after the monolith’s functionalization by thiol groups by immersion of monoliths in the MPTMS solution and its further hydrolysis of alkoxy groups in water for 24 h. As seen, this functionalization method led to a much larger decrease in *S*_BET_ of the M(P123) than of M(F127), suggesting the formation of a larger amount of micropores in the former material. The sorbent M(F127)–SH exhibits relatively narrow pore size distribution (Figure 2), which may be an indication that the –SH groups on the surface blocked the smaller pores. In fact, the sorbent M(P123)–SH has a much smaller total pore volume (0.69 vs. 2.26 m^3^ g^−1^) and a larger pore diameter than M(F127)–SH (9.6 vs. 7.6 nm).

**Figure 3:**
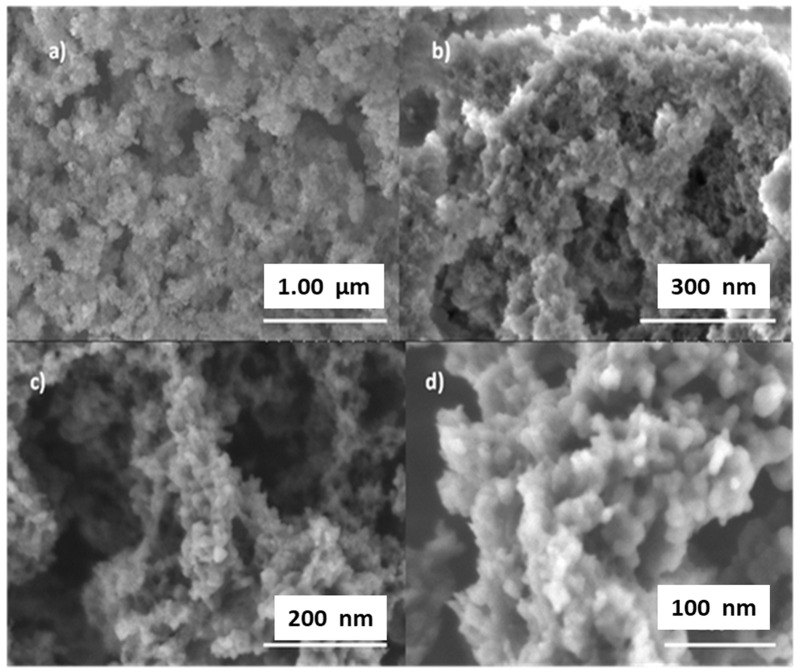
SEM images of the M(P123)–SH sorbent at different magnitudes (X): (**a**), X = 200,000, (**b**), X = 150,000 and (**c**,**d**), X = 300,000, evidencing the multimodal morphology and wormhole pores.

**Figure 4:**
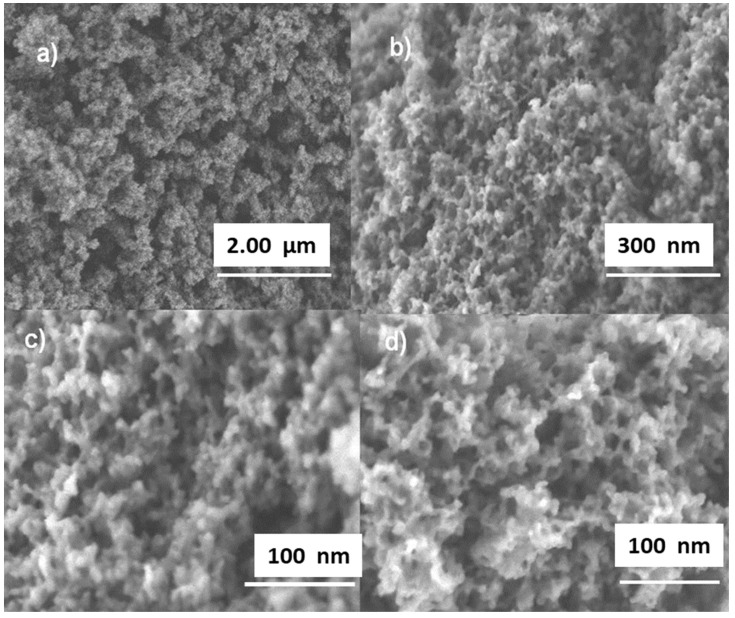
SEM image of the material M(F127)–SH at different magnifications (X): (**a**) X = 40,000, (**b**) X = 50,000, (**c**) X = 200,000 and (**d**) X = 350,000, evidencing its multimodal morphology.

The information obtained from the pore size distribution was confirmed by the SEM characterization of the M(P123)–SH sorbent (Figure 3). The different pore sizes observed in image Figure 3a evidence the macroporosity of this sorbent forming a wormhole-like structure that is characteristic of a hierarchical material [53,54]. In Figure 3b (at 150,000 magnification), a porosity with an average pore diameter in the range of 50–60 nm is shown. In the same analysis area, at 300,000 magnifications, the monolith shows a porosity with a pore diameter in the range of 10–4 nm (Figure 3c,d). Considering the study of Boutros et al. [55] and Stevens et al. [56], the micropores of the sorbents M(P123) and M(F127) can be formed during their synthesis, due to the penetration of the more hydrophilic EO chains of the tri-block copolymer into the silica wall. The whiteness is due to the porous nature of the monoliths. The comparison of SEM images of both thiol-functionalized monoliths shown in Figure 3 and Figure 4, respectively, clearly shows that the M(P123)–SH monolith exhibits a higher amount of macro- and mesopores than its M(F127)–SH counterpart.

Figure 4a–d shows SEM images of the M(F127)–SH sorbent, illustrating the presence of pores in the ranges of 50–200 nm (Figure 4a), 10–50 nm (Figure 4b), 6–40 nm (Figure 4c) and 2–10 nm (Figure 4d), thus confirming the hierarchical pore structure of this material. Compared to M(P123)–SH (Figure 3), M(F127)–SH exhibits smaller pores and a denser silica structure framework.

#### 3.1.2. Framework Vibration

**Figure 5:**
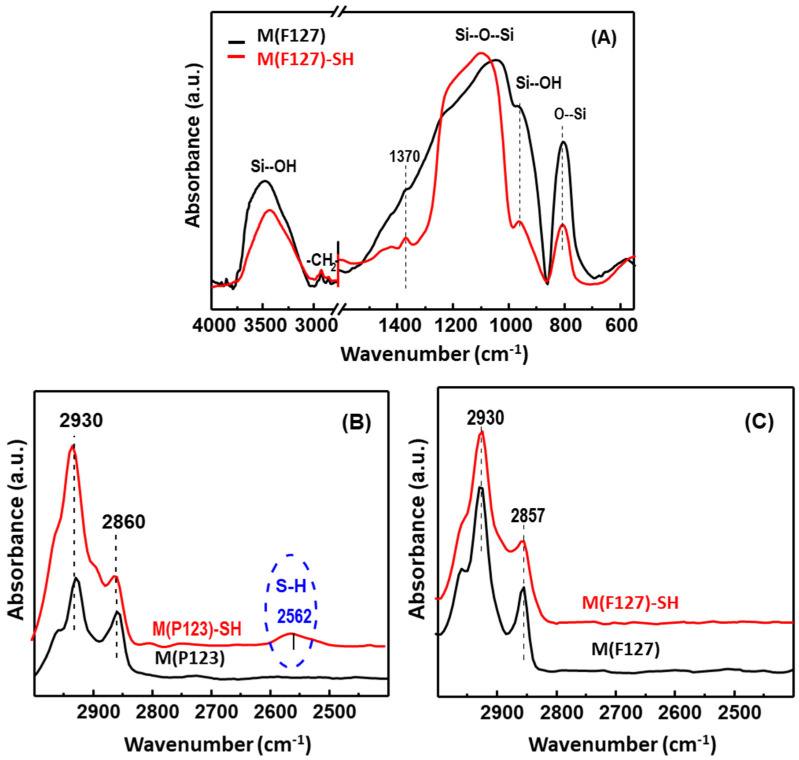
FTIR-KBr spectra of hierarchical monolithic silica adsorbents before and after functionalization with propylthiol groups showing the stretching vibration region of the Si–O–Si bond of the silica structure (**A**), and the stretching vibration region of the thiol groups (**B**,**C**).

The existence of thiol groups in the hierarchical monoliths was confirmed by studying their frame-vibrational region by FTIR-KBr spectroscopy. As an example, Figure 5A shows the FTIR spectra of M(F127)–SH and M(F127)–SH, where a typical silica (SiO_2_) spectrum is observed. Absorption bands in the range 1000–1150 cm^−1^ and a shoulder around 1150–1250 cm^−1^ are attributed to the symmetric and antisymmetric stretching vibrations of the Si–O–Si bond of the silica structure, respectively [57], while the absorption bands around 967 cm^−1^ and around 800 cm^−1^ can be assigned to the non-condensed silanol (Si–OH) groups and symmetric stretching vibration from Si–O bonds, respectively [58]. In addition, all samples show the 1635 cm^−1^ band belonging to free molecular H_2_O (not shown here). This assignment is consistent with the broad band centered at 3442 cm^−1^, which is a characteristic of physically adsorbed molecular water interacting by H-bonding with silanol groups. For both thiol-functionalized adsorbents, the bands in the range 2870–3000 cm^−1^ can be attributed to the (–CH_2_–) bond of the thiol-propyl groups of the adsorbents, as well as to the (–CH_3_) and (–CH_2_–) organic groups of the template (P123 and F127). In fact, the absorption band around 1370 cm^−1^ evidenced the presence of the polymer residue in both functionalized and non-functionalized adsorbents. Most notably, M(P123)–SH shows a band around 2562 cm^−1^ attributed to the stretching vibrations of the thiol groups (υ(SH)) (Figure 5B). The presence of this band confirmed the successful functionalization of the monolithic silica adsorbent M(P123)–SH by grafting with propylthiol groups. In contrast to M(P123)–SH, M(F127)–SH did not show the band around 2562 cm^−1^, suggesting that this adsorbent may have a lower amount of thiol groups than its counterpart.

#### 3.1.3. Thermal Stability of –SH Groups

**Figure 6:**
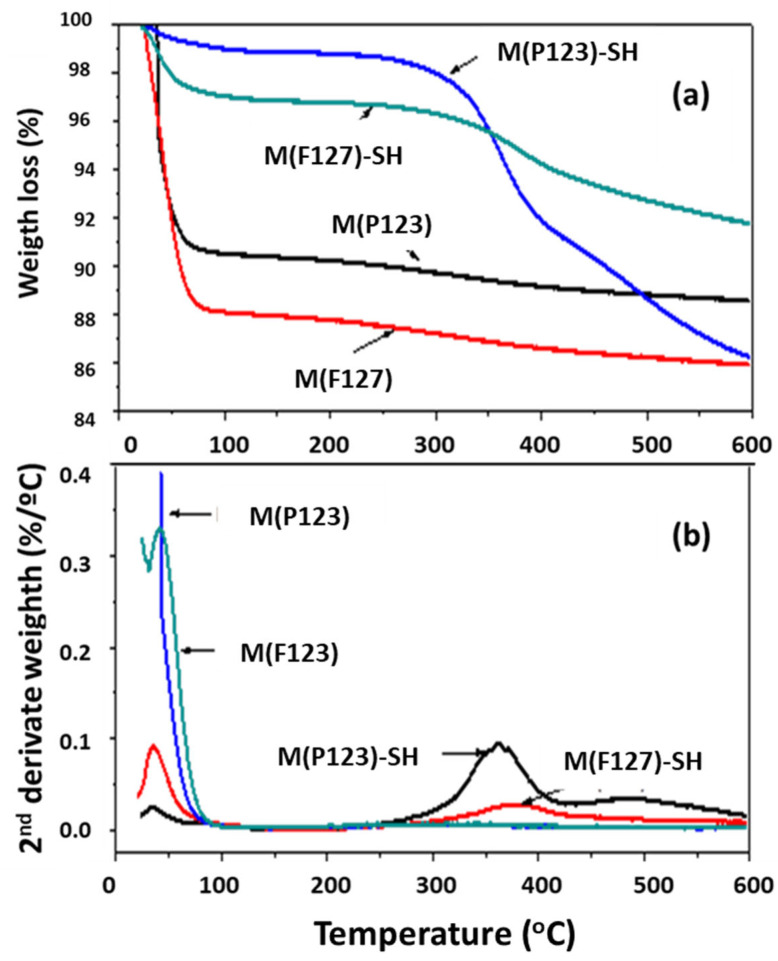
TGA (**a**) and DTG (**b**) curves of the hierarchical silica monoliths before and after functionalization with –SH groups.

The thermal stability of the thiol groups on the surface of M(P123)–SH and M(F127)–SH silica monoliths was evaluated by thermogravimetric analysis (TGA-DTG) by heating the sorbents from room temperature to 600 °C. Figure 6a,b compares the TGA and DTG profiles, respectively, of the M(P123) and M(F127) sorbents before and after functionalization with thiol groups. The inflection points observed in the DTG curves of pure M(P123) and M(F127) monoliths at 45 °C and 50 °C, respectively, are due to dehydration of the pure silica material [59]. In this low-temperature region, the weight loss of the non-functionalized and pure samples is similar: 9.1% and 12.2% for M(P123) and M(P123)–SH, respectively, and 12.0% and 9.2% for M(F127) and M(F127)–SH, respectively

In contrast to the non-functionalized monoliths, the DTG curves of the thiol-functionalized samples show peaks at 364 °C and 380 °C related to the decomposition of the mercapto groups of the M(P123)–SH and M(F127)–SH monoliths, respectively (Figure 6b). It is noteworthy that the latter sample shows a higher stability of the thiol groups than the former, as deduced from the higher decomposition temperature of the –SH groups and a lower weight loss in the high-temperature region (Figure 6a). In fact, in contrast to M(F127)–SH, the M(P123)–SH sample shows a small peak around 507 °C, indicating the decomposition of its methylene groups (Figure 6b). In summary, regardless of the type of copolymer used for the synthesis of the silica matrix, the –SH groups exhibit high thermal stability (above 300 °C), suggesting their strong chemical bonding with the –OH groups of the silica matrix.

### 3.2. Adsorption Experiments

**Figure 7:**
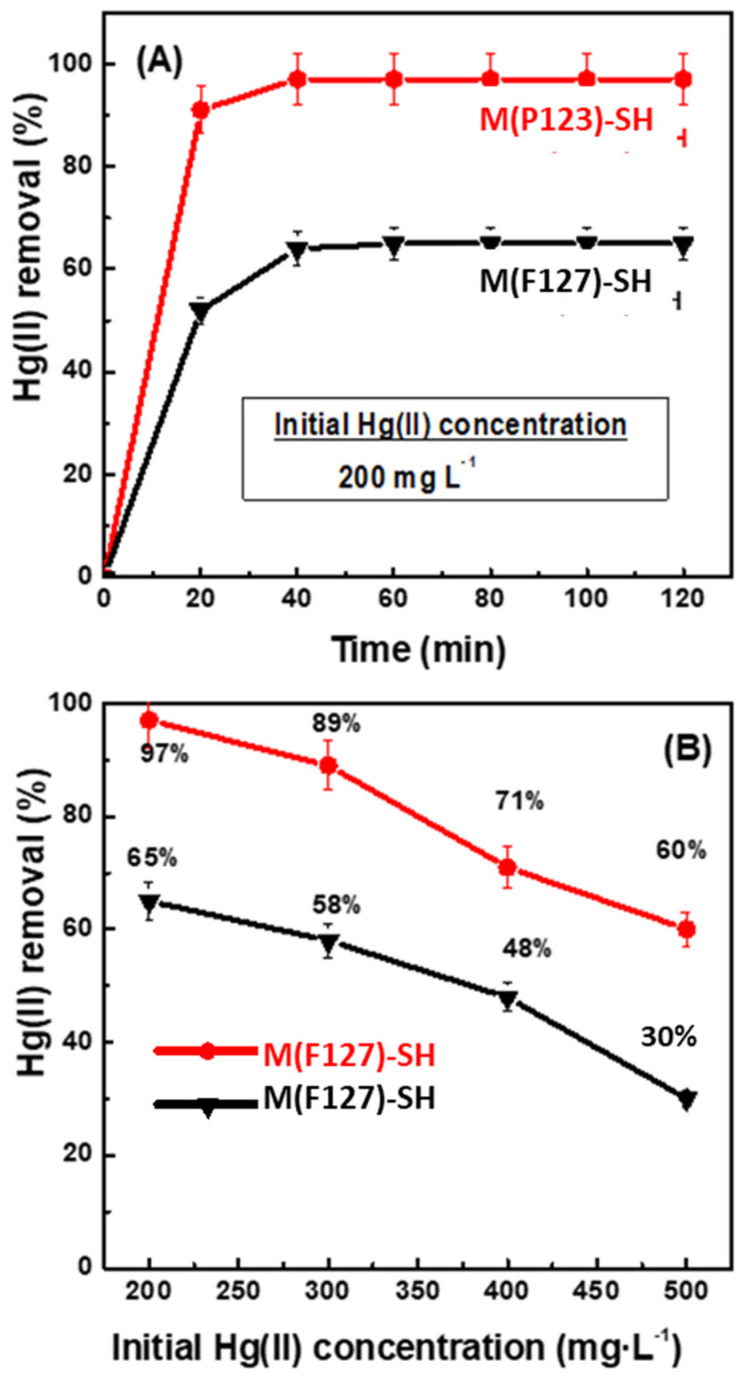
(**A**) Effect of adsorption time on Hg(II) ion removal efficiency, and (**B**) effect of initial Hg(II) concentration on Hg(II) removal for M(P123)–SH and M(F127)–SH.

Atomic absorption spectroscopy was employed to study the ability of the synthesized silica monoliths to remove Hg(II) ions from aqueous medium. Since the improvement of adsorption capacity is expected after their functionalization with –SH groups [9,12,13,14,15,16,17,18,19,20,21,22,23,24,25,26,27,28,29,30,31,32,33,34,35,36,37,38,39], the effect of using Pluronic P123 and F127 co-polymers in the synthesis of silica matrix was investigated for the thiol-functionalized samples. Figure 7A,B shows the effect of adsorption time on the Hg(II) ion removal efficiency of M(P123)–SH and M(F127)–SH sorbents and their mercury adsorption ability as a function of the initial concentration of Hg (II) ions in the aqueous solution, respectively. As can be seen, for both sorbents, the equilibrium Hg (II) ion concentration was reached after 40 min of contact time (Figure 7A). Under the same adsorption conditions (pH 3.5, 28 °C and contact time of 1 h), the adsorption capacity of M(P123)–SH was much higher than that of its counterpart M(F127)–SH. For both thiol-functionalized sorbents, the percentage of mercury removal decreased with an increase of the initial Hg(II) concentration in water from 200 to 500 mg·L^−1^, suggesting saturation of the –SH groups (Figure 7B). Using the aqueous solution with the lowest mercury concentration (200 mg·L^−1^), M(P123)–SH and M(F127)–SH show a percentage of Hg(II) removal of about 97% and 65%, respectively. This means that, after 1 h of contact, the concentration of Hg(II) ions in solution using the M(P123)–SH sorbent decreased from 200 to 6 mg·L^−1^. For solutions with Hg(II) ion concentration of 300, 400 and 500 mg·L^−1^, the percentage of Hg(II) that was removed by using the M(P123)–SH sorbent was 89%, 71% and 60%, respectively (267, 284 and 300 mg·L^−1^, respectively).

The adsorption conditions were contact time 60 min, pH 3.5, 28 °C and 0.1 g of sorbent.

In order to compare the adsorption capacity of the synthetized sorbents with those reported in the literature, their adsorption capacity was calculated by using following equation:

*Q* = [*C*_0_ ·*V* −*C*_f_ *V*]/*m* (1)

where *Q* (mg g^−1^) is sorbent adsorption capacity; *C_0_* (mg L^−1^) and *C*_f_ (mg L^−1^) are the initial and final Hg(II) ions concentration, respectively; and *m (g)* and *V* (L) are the mass of sorbent and volume of the Hg(II) ion solution, respectively.

Table 2 compares the adsorption capacity of our synthesized sorbents with those reported in the literature for macroporous/mesoporous sorbents. At similar batch adsorption conditions, our best M(P123)–SH sorbent exhibited a higher adsorption capacity than those reported for thiol functionalized mesoporous materials, such as SBA–15 [13,14] and MCM–41 [15], having a high surface area and abundant macro/mesoporosities (Table 2). This means that silica monoliths having hierarchical porosity offer promising sorption capacity for the removal of Hg(II) ions from wastewater. The results are in good agreement with the study of Arsuaga et al. [29], showing that, even at a very low concentration of Hg(II) ions in aqueous solution, the adsorbent in pellet form was more effective for mercury removal than the same adsorbent in powder form. Representation of the isotherms as the amount of Hg adsorbed on the chylibium (expressed as millimoles per gram of adsorbent), versus the equilibrium mercury concentration in the liquid phase, expressed as milligrams per liter) allows the calculation of the maximum mercury capacity. For the sorbents M(P123)–SH and M(F127)–SH, the maximum mercury adsorption capacity was 12.2 and 7.5 mmol per gram of adsorbent, respectively. The latter adsorbent showed an adsorption capacity a little higher than that reported by Aguado et al. [34] for propylthiol-functionalized SBA–15 prepared by co-condensation (4.1 mmol of Hg(II) per gram of adsorbent). The extremely high sorption capacity for Hg(II) ions removal from aqueous solution (∼500 mg g^−^) demonstrated an adsorbent having the MoS_4_^−2^ ion intercalated into Mg-Al layered double hydroxide [35]. The adsorption was exceptionally rapid and highly selective (97.3% removal within 5 min).

The shape of the monolith could also explain why, in addition to its poorly ordered structure, the M(P123)–SH monolith showed very good adsorption capacity. In this regard, there are works indicating that the ordering of mesoporous materials is not of great importance if the solids have a high specific surface area for thiol group attachment [60]. This was confirmed in this work because M(P123)–SH proved to be an effective adsorbent for Hg(II) removal. However, there is also work indicating that a high degree of incorporation of thiol groups and their good availability could be archived only with highly ordered materials [36,37]. Therefore, further work is needed to clarify this point.

Considering the literature reports [28,32], a good regeneration of the synthesized monolithic adsorbents could be assumed. Since the reuse of thiol-functionalized siliceous materials is a complex problem, it will be our issue in the following work. The reusability of adsorbents is highly dependent on the type and concentration of the regenerant used [33], and the results presented in the literature are contradictory. For example, on the one hand, it was reported that HCl shows appreciable ability to regenerate thiol-functionalized SBA-15 adsorbent, while the use of nitric acid proved to be inefficient [28,38]. On the other hand, regeneration of SiO_3_–SH microspheres with hydrochloric acid was found to be more effective than their regeneration with nitric acid [33]. Other complexing compounds, such as KBr, KSCN, (NH_2_)_2_CS and HBr, are also effective for the desorption of significant amounts of adsorbed mercury, and the best results were obtained by using HBr [28]. In fact, after five cycles of Hg(II) adsorption at pH 8 and regeneration with HBr, the adsorption capacity of SBA-15-SH decreased by approximately 18.7%, indicating the good reusability and hydrothermal stability of this adsorbent [32]. 

**Figure 8:**
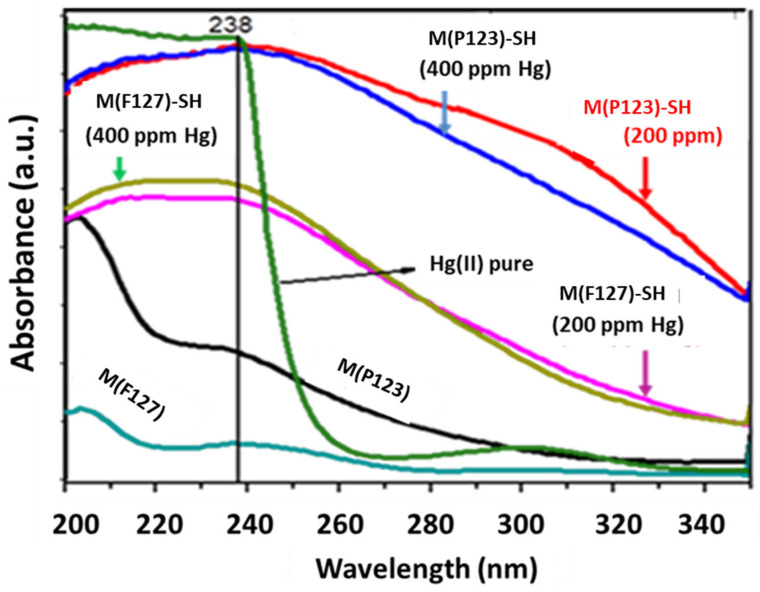
UV–Vis spectra of the sorbents M(P123)–SH and M(F127)–SH after adsorption of Hg(II) from aqueous solutions containing 200 and 400 mg/L Hg(II).

The confirmation of Hg(II) adsorption on both M(P123)–SH and M(F127)–SH was obtained by using UV–Vis and XPS characterization. Figure 8 shows the UV–Vis adsorption spectra of both sorbents before and after Hg (II) adsorption from 200 and 400 mg/L water solutions. As seen in this figure, prior to adsorption experiments, both substrates show a band at 238 nm and 195 nm, due to C–S chromospheres at the n-σ* transition of 3-mercaptopropyl-trimethoxysilane [61]. After Hg(II) adsorption from 200 and 400 mg/L solutions, the high adsorption in the spectral region of 200–350 nm was observed, thus confirming Hg(II) adsorption. The ligand-to-metal charge-transfer transitions are intuited at about *λ* 280 nm and 300 nm [62]. It is noteworthy that the sorbent prepared with the P123 copolymer was more efficient for Hg(II) removal than its M(F127)–SH counterpart, as deduced from the higher intensity of both bands of M(P123)–SH [63]. Therefore, for both thiol-functionalized adsorbents, the presence of ligand-to-metal charge-transition bands confirmed Hg(II) adsorption on the propylthiol groups. In contrast to M(P123)–SH, an increase in the concentration of Hg(II) ions in solution from 200 to 400 ppm does not change the intensity of the band in the region 280–350 nm, confirming its lower Hg(II) adsorption capacity. In the case of M(P123)–SH, the decrease in the intensity of this band after adsorption of Hg(II) from an aqueous solution of 400 mg·L^−1^ concentration could be explained by considering the possible electrostatic repulsion force between the formed S–Hg^+^ species and the Hg^2+^ ions still present in the aqueous solution.

The surface chemical analysis of the M(P123)–SH and M(F127)–SH monoliths before and after removal of Hg(II) ions from 200 and 400 mg L^−1^ aqueous solutions was investigated by X-ray photoelectron spectroscopy analysis. The binding energies of Si 2p, O 1s and Hg 4f core electrons of both sorbents are compiled in Table 3. The peaks at ca. 532.9 eV and 103.3 eV are due to O^2−^ and Si^4+^ ions, respectively, forming –Si–O–Si– bonds in the silica matrix of the monolith [23]. Noticeably, the adsorption of Hg from 400 mg L^−1^ solution led to the reduction of Hg(II) to Hg (0), as evidenced by the presence of the peaks with BE value 99.9 eV. Simultaneously, S 2p_3/2_ peaks at 162.2, 163.4 and 167.9 eV are observed, indicating the simultaneous reduction and oxidation of the −SH groups [64].

As expected, two peaks at 101.0 eV (Hg 4f_7/2_) and 104.4 eV (Hg 4f_5/2_) derived from spin–orbit splitting are observed, indicating the formation of Hg–O bonds [65]. Similar peaks are presented by the M(F127)–SH sorbent (spectra not shown here). For both sorbents, no evidence of Hg precipitation as hydroxides or carbonates was observed.

The best sorbent M(P123)–SH was synthesized by using the P123 polymer, having a smaller number of EO chains than the F127 polymer. The hierarchical porous structure of the M(P123)–SH monolith allows for easier transport of Hg(II) ions to the adsorption sites, leading to improved adsorption capacity. The lower efficiency of M(F127)–SH with respect to M(P123)–SH can be explained by considering a more difficult access of Hg(II) to the thiol groups located in the less accessible positions and its more hydrophilic character originating from a higher number of EO chains of the F127 polymer [20,53]. The results presented in this work are in agreement with recent findings indicating that the macroporosity of the sorbent has a positive effect on its adsorption efficiency [1,4,5,21].

Successful functionalization of the monolithic adsorbents with thiol groups was confirmed by FTIR-KBr, UV–Vis and XPS. The FTIR-KBr spectra of the most efficient adsorbent (M(P123)–SH) show a peak around 2562 cm^−1^ attributed to the stretching vibrations of the thiol groups (υ(SH)) (Figure 5B). The presence of this peak can be taken as evidence of S–H involvement in the reaction mechanism. In contrast to M(F127)–SH, thiol groups are still present on the surface of M(P123)–SH, suggesting that this adsorbent may have a higher amount of thiol groups than its counterpart. On the other hand, after adsorption of Hg(II) ions, the XPS spectra of the M(P123)–SH adsorbent show only a pair of Hg 4f_5/2_ and Hg 4f_7/2_ peaks, confirming the presence of Hg(II) ions. 

With regard to the mechanism of elimination of Hg(II) from aqueous solution, UV–Vis and XPS characterizations of the monoliths after Hg(II) adsorption confirmed the trapping of Hg(II) by both monolithic silica adsorbents. After adsorption of Hg(II) ions from a low concentration solution (200 mg/L), the XPS spectra of the M(P123)–SH adsorbent show only a pair of Hg 4f_5/2_ and Hg 4f_7/2_ peaks, confirming the presence of Hg(II) ions. 

Being a soft Lewis acid, Hg(II) has a strong affinity for soft Lewis base thiol-functional groups. By applying Pearson’s hard and soft acids and bases (HSAB) theory [66,67], an interaction between –SH groups and Hg(II) ions led to formation of the stable complex. Considering the negative zeta potential of silica adsorbent at pH 3.5, originating from the presence of silanol groups [33], we see that an electrostatic interaction occurs between the negatively charged surface of silica adsorbents and positively charged Hg^2+^ ions [66,67]. Such an interaction was recently confirmed by Liang and Zou for non-porous silica nanospheres tested for Hg(II) removal in the pH range of 2.5 and 5.5 [33]. At pH 3.5, the aqueous solution mainly contains both Hg^2+^ and HgOH^+,^ which, upon interaction with thiol groups, leads to the formation of S–Hg^+^ species [33]. Therefore, the electrostatic repulsive force between surface S–Hg^+^ species and the Hg^2+^ ions present in the aqueous solution define the limit of the adsorption capacity of the adsorbent.

The high adsorption capacity (132.6 mg·g^−1^) for mercury ions’ removal from aqueous solution and soft acidic conditions (pH 5) was reported for the nanoporous tetrasulfide-functionalized fibrous silica by Marjani and Mohammadi [11]. Considering the N_2_-physisorption and SEM data, the superior adsorption properties of M(P123)–SH can be ascribed to its hierarchical pore structure. For the solution concentrations higher than 200 mg·L^−1^, the adsorption properties of M(P123)–SH could be improved by optimizing the adsorption conditions, such as temperature [68], contact time [69] or pH [70]. Interestingly, the adsorption capacity of the best M(P123)–SH monolith is much lower when compared to the Hg(II) adsorption capacity of the non-porous SiO_2_–SH microspheres, which were prepared at a low temperature, using a one-step sol–gel method employing ultrasonic treatment (197 vs. 274.1 mg·g^−1^). Using the Langmuir model, the estimated maximum adsorption capacity of the non-porous SiO_2_–SH was 377.36 mg g^−1^ [33]. In general, high-efficiency adsorbents recently presented in the literature show high adsorption capacity for the removal of heavy metals from wastewater [33,37]. In general, their high efficiency is linked to easier access of Hg(II) ions to thiol groups owning their hierarchical pore structure with high density of functional groups [71]. In some cases, their adsorption capacity is even higher than that of commonly used adsorbents. For example, the high efficiency of 99.9% for removal of traces of Hg(II) from drinking water was reported by Ren et al. for a covalent organic polymer having hierarchical pore structure and a large amount of sulfhydryl sites [71]. The polymer was prepared via one-pot diazo-coupling reaction between ETTA and 1,4-benzenedi thiol. Very high Hg(II) uptake capacity (478.47 mg/g) was reported for magnetic mesoporous silica/chitosancomposite by He et al. [72]. Recently, extensive reviews of newly developed sorbents were presented by Modak et al [17] and Da’na [18]. In view of recent findings [17,18], we conclude that the monolithic silica materials synthesized in this work could also have practical applications as heterogeneous catalysts, for gas storage or as sensors detecting Hg(II) ions [60].

## 4. Conclusions

The adsorption study indicates that the hierarchical silica monolith synthesized with Pluronic P123 co-polymer and modified with propylthiol groups by grafting could be a promising adsorbent for total removal of Hg(II) ions from wastewater. The best monolith functionalized with thiol groups exhibited a high surface area and pore diameters suitable for the contact of Hg(II) ions with thiol groups. The hierarchical structure provides thermal stability to the thiol groups (above 300 °C), as this is an important feature for its potential application as an adsorbent. The M(P123)–SH sorbent showed almost total adsorption of Hg ions (97%) from aqueous solution with a concentration of 200 mg L^−1^, which is much higher adsorption than other silica-based adsorbents tested under similar conditions. We concluded that the hierarchical pore structure of the monoliths prepared with P123 polymer favors the accessibility of Hg(II) ions to the grafted –SH groups. In addition to their use as adsorbents, the monolithic silica materials synthesized in this work could also have practical application as heterogeneous catalysts, for gas storage or as Hg(II) ion detection sensors.

## Figures and Tables

**Table 1:** Textural properties of the hierarchical monoliths before and after of functionalization with −SH groups (from N_2_ physisorption at −196 °C).

Sorbent	S_BET_ (m^2^/g)	d (nm)	V (m^3^/g)
M(P123)	530	3.4	0.42
M(P123)–SH	281	9.6	0.69
M(F127)	527	3.4	0.36
M(F127)–SH	515	7.9	2.26

**Table 2:** Comparison of the sorption capacity of the silica-based sorbents reported in the literature.

Sorbent	Initial Hg(II) Concentration (mg L^−1^)	Adsorption Conditions pH/T/Time	Sorption Capacity (mg/g)	Reference
M(P123)–SH ^(a)^	200	pH 3.5; 28 °C; 1 h	194.0	This work
M(F127)–SH ^(a)^	200	pH 3.5; 28 °C; 1 h	70.0	This work
TS–KCC–1 ^(b)^	200	pH 5; 25 °C; 40 min	132.6	[12]
MPTMS–SMs ^(c)^	160	pH 7.5; 30 °C; 1500 min	62.3	[13]
FTU–SBA–15 ^(d)^	200	pH 6; 25 °C;	122.4	[14]
MBT–SBA–15 ^(e)^	100	pH 6; 25 °C; 4 h	48.1	[15]
MBT–MCM–41 ^(f)^	100	pH 6; 25 °C; 4 h	42.1	[16]
SiO_2_–SH ^(g)^	150	pH 5.5; 20 °C, 24 h	274.1	[34]
MTZ–MCM–41 ^(h)^	647	pH 6; 25 °C, 4 h	140.4	[18]
MP–MCM–41 ^(i)^	100	pH 6; 25 °C; 4 h	38.1	[18]
MP–SBA–15 ^(j)^	100	pH 6: 25 °C; 4 h	20.0	[18]
MoS_4_–LDH ^(j)^	1162	pH 4.11; 20 °C; 6 h	500	[35]

^(a)^ Silica monolith modified with 3-mercaptiopropyltrimethylosilane (MPTMS); ^(b)^ tetrasulfide-functionalized fibrous silica; ^(c)^ 3-mercaptopropyl trimethoxysilane; ^(d)^ 1-furoyl thiourea-functionalized SBA15; ^(e)^ 2-mercaptobenzothiazol-functionalized SBA–15(MCM–41); ^(f)^ Non-porous SiO_2_–SH microspheres modified with MPTMS; ^(g)^ 2-mercaptothiazoline-functionalized MCM–41; ^(h)^ 2-mercaptopyridine-functionalized MCM–41; ^(i)^ 2-mercaptopyridine-functionalized SBA–15; ^(j)^ MoS_4_^−2^ ion intercalated into Mg–Al layered double hydroxide.

**Table 3:** Binding energies (eV) of core electrons of the M(P123)–SH adsorbent before and after adsorption of Hg(II) ions.

XPS Core Level	Before Hg(II) Adsorption	After Hg(II) Adsorption 200 mg/L	After Hg(II) Adsorption 400 mg/L
Si 2p	103.3	103.3	103.3
O 1s	532.9	532.8	532.9
Hg 4f_7/2_	-	101.0	101.0
Hg 4f_5/2_	-	104.4	104.4

## Data Availability

Data is contained within the article.

## References

[1] Ren Y., Yu H., Hua M., Lv L., Zhang W. (2021). Highly efficient and selective Hg(II) removal from water by thiol-functionalized MOF–808: Kinetic and mechanism study. Chem. Eng. J..

[2] Šćiban M., Klašnja M., Škrbić B. (2006). Modified softwood sawdust as adsorbent of heavy metal ions from water. J. Hazard. Mater..

[3] Bernard E., Jimoh A. (2013). Adsorption of Pb, Fe, Cu, and Zn from industrial electroplating wastewater by orange peel activated carbon. Int. J. Eng. Appl. Sci..

[4] Xia N.N., Zhang B., Hu Z.H., Kong F., Xu G., He F. (2021). A biomass-assembled macro/mesoporous nano-scavenger for Hg ion trapping. New J. Chem..

[5] Ho K.Y., Yeung K.Y., McKay G. (2003). Selective Adsorbents from Ordered Mesoporous Silica. Langmuir.

[6] Xue X., Li F. (2008). Removal of Cu(II) from aqueous solution by adsorption onto functionalized SBA–16 mesoporous silica. Micropor. Mesopor. Mater..

[7] Zeng W., Bai H. (2015). Adsorption of acetone vapors by SBA–16 and MCM–48 synthetized from rice husk ash. Int. J. Environ. Chem. Ecol. Geol Geophys. Eng..

[8] Shah A.T., Din M.I., Kanwal F.N., Mirza M.L. (2015). Direct synthesis of mesoporous sieves of Ni–SBA–16 by internal pH adjustment method and its performance for adsorption of toxic Brillant Green dye. Arabian. J. Chem..

[9] Lesaint C., Frébault F., Delacôte C., Lebeau B., Marichal C., Walcarius A., Patarin J. (2005). Synthesis and characterization of mesoporous silicas functionalized by thiol groups, and application as sorbents for mercury (II). Stud. Surf. Sci. Catal..

[10] Han S.C., Sujandi S., Park S.E. (2005). Functionalization of SBA–16 mesoporous materials with cobalt(III) cage amine complex. Bull. Korean Chem. Soc..

[11] Marjani A., Mohammadi R.K. (2021). Synthesis of novel adsorbent based on tetrasulfide-functionalized fibrous silica KCC–1 for removal of Hg(II) cations. Sci. Rep..

[12] Saman N., Johari K., Mat H. (2014). Adsorption characteristics of sulfur-functionalized silica microspheres with respect to the removal of Hg(II) from aqueous solutions. Ind. Eng. Chem. Res..

[13] Mureseanu M., Reiss A., Cioatera N., Trandafr L., Hulea V. (2010). Mesoporous silica functionalized with 1-furoyl thiourea urea for Hg(II) adsorption from aqueous media. J. Hazard. Mater..

[14] Pérez-Quintanilla D., del Hierro I., Fajardo M., Sierra I. (2006). Preparation of 2-mercaptobenzothiazole-derivatized mesoporous silica and removal of Hg(II) from aqueous solution. J. Environ. Monit..

[15] Pérez-Quintanilla D., del Hierro I., Fajardo M., Sierra I. (2006). 2-Mercaptothiazoline modifed mesoporous silica for mercury removal from aqueous media. J. Hazard. Mater..

[16] Pérez-Quintanilla D., Del Hierro I., Fajardo M., Sierra I. (2006). Mesoporous silica functionalized with 2-mercaptopyridine: Synthesis, characterization and employment for Hg(II) adsorption. Micropor. Mesopor. Mater..

[17] Modak A., Bhanja P., Selvaraj M., Bhaumik A. (2020). Functionalized porous organic materials as efficient media for the adsorptive removal of Hg(II) ions. Environ. Sci. Nano.

[18] Da’na E. (2017). Adsorption of heavy metals on functionalized-mesoporous silica: A review. Micropor. Mesopor. Mater..

[19] Smått J.H., Schunk S., Linde’n M. (2003). Versatile Double-Templating synthesis route to silica monoliths exhibiting a multimodal hierarchial porosity. Chem. Mater..

[20] Higgins S., Kennard R., Hill N., DiCarlo J., DeSisto W.J. (2006). Preparation and characterization of non-ionic block co-polymer templated mesoporous silica membranes. J. Membr. Sci..

[21] Hristov P., Yoleva A., Djambazov S., Chukovska I., Dimitrov D. (2012). Preparation and characterization of porous ceramic membranes for microfiltration from natural zeolite. J. Univ. Chem. Techn. Metall..

[22] Kumar P., Guliants V.V. (2010). Periodic mesoporous organic-inorganic hybrid materials: Applications in membrane separations and adsorption. Micropor. Mesopor. Mater..

[23] Tsai C.Y., Tam S.Y., Lu Y., Brinker C.J. (2000). Dual-layer asymmetric microporous silica membranes. J. Membr. Sci..

[24] Hernández-Morales V., Nava R., Acosta-Silva Y.J., Macías-Sánchez S.A., Pérez-Bueno J.J., Pawelec B. (2012). Adsorption of lead (II) on SBA–15 mesoporous molecular sieve functionalized with –NH_2_ groups. Micropor. Mesopor. Mater..

[25] Heidari A., Younesi H., Mehraban Z. (2009). Removal of Ni(II), Cd(II), and Pb(II) from a ternary aqueous solution by amino-functionalized mesoporous and nano mesoporous silica. Chem. Eng. J..

[26] Kresge C.T., Leonowicz M.E., Roth W.J., Vartuli J.C., Beck J.S. (1992). Ordered mesoporous molecular sieves synthesized by liquid-crystal template mechanism. Nature.

[27] Peter C.A., Alberius K.L., Frindell R.C., Hayward E.J., Kramer G.D., Stucky B.F., Chmelka B.F. (2002). General predictive syntheses of cubic, hexagonal and lamellar silica and titania mesostructured thin films. Chem. Mater..

[28] Arencibia A., Aguado J., Arsuaga J.M. (2010). Regeneration of thiol-functionalized mesostructured silica adsorbents of mercury. Appl. Surf. Sci..

[29] Arsuaga J.M., Aguado J., Arencibia A., López-Gutiérrez M.S. (2014). Aqueous mercury adsorption in a fixed bed column of thiol functionalized mesoporous silica. Adsorption.

[30] Aguado J., Arsuaga J.M., Arencibia A. (2010). Heavy metals removal from water by adsorption on propylthiol-functionalised mesoporous silica obtained by co-condensation. Int. J. Environ. Technol. Manag..

[31] Lourenço M.A.O., Figueira P., Pereira E., Gomes J.R.B., Lopes C.B., Ferreira P. (2017). Simple, mono and bifunctional periodic mesoporous organosilicas for removal of priority hazardous substances from water: The case of mercury(II). Chem. Eng. J..

[32] Shen Y., Jiang N., Liu S., Zheng C., Wang X., Huang T., Guo Y., Bai R. (2018). Thiol functionalization of short channel SBA–15 through a safe, mild and facile method and application for the removal of mercury (II). J. Environ. Chem. Eng..

[33] Ling R., Zou H. (2020). Removal of aqueous Hg(II) by thiol-functionalized nonporous silica microspheres prepared by one-step sol–gel method. RSC Adv..

[34] Aguado J., Arsuaga J.M., Arencibia A. (2008). Infuence of synthesis conditions on mercury adsorption capacity of propylthiol functionalized SBA–15 obtained by co-condensation. Mesopor. Mesopor. Mater..

[35] Ma L., Wang Q., Islam S.M., Liu Y., Ma S., Kanatzidis M.G. (2016). Highly selective and efficient removal of heavy metals by layered double hydroxide intercalated with the MoS_4_^2−^ ion. J. Am. Chem. Soc..

[36] Aguado J., Arsuaga J.M., Arencibia A. (2009). Adsorption of aqueous mercury (II) on propylthiol-functionalized mesoporous silica obtained by cocondensation. Ind. Engin. Chem. Res..

[37] Lee B., Kim Y. (2001). Synthesis of functionalized porous silicas via templating method as heavy metal ion adsorbents: The introduction of surface hydrophilicity onto the surface of adsorbents. Micropor. Mesopor. Mater..

[38] Sajjadi S.A., Mohammadzadeh A., Tran H.N., Anastopoulos I., Dotto G.L., Lopičić Z.R., Sivamani S., Rahmani-Sani A., Ivanets A., Hosseini-Bandegharaei A. (2018). Efficient mercury removal from wastewater by agent. J. Environ. Manag..

[39] McCool B.A., Hill N., Di Carlo J., De Sisto W.J. (2003). Synthesis and characterization of mesoporous silica membranes pistachio wood wastes-derived activated carbon prepared by chemical activation using a novel activating via dip-coating and hydrothermal deposition techniques. J. Membr. Sci..

[40] Smarsly B., Xomeritakis G., Yu K., Liu N., Fan H., Assink R.A., Drewien C.A., Ruland W., Brinker C.J. (2003). Microstructural characterization of polystyrene-block-poly(ethylene oxide)-Templated silica films with cubic-oredere spherical mesopores. Langmuir.

[41] Boissiere C., Martines M.U., Larbot A., Prouzet E. (2005). On the specific filtration mechanism of mesoporous silica membrane, prepared with non-connecting parallel pores. J. Membr. Sci..

[42] Nakanishi K.J. (1997). Pore Structure control of silica gels based on phase separation. Porous Mater..

[43] Minakuchi H., Nakanishi K., Soga N., Ishizuka N., Tanaka N. (1996). Octadecylsilylated porous silica rods as separation media for reversed-phase liquid chromatography. Anal. Chem..

[44] Nakamura N., Takahashi R., Sato S., Sodesawa T., Yoshida S. (2000). Ni/SiO_2_ catalyst with hierarchical pore structure prepared by phase separation in sol-gel process. Phys. Chem. Chem. Phys..

[45] Deshmukh M.A., Shirsat M.D., Ramanaviciene A., Ramanavicius A. (2018). Composites Based on Conducting Polymers and Carbon Nanomaterials for Heavy Metal Ion Sensing (Review). Crit. Rev. Anal. Chem..

[46] Deshmukh M.A., Celiesiute R., Ramanaviciene A., Shirsat M.D., Ramanavicius A. (2018). EDTA_PANI/SWCNTs Nanocomposite Modified Electrode for Electrochemical Determination of Copper (II), Lead (II) and Mercury (II) Ions. Electr. Acta.

[47] Wang J., Dong B., Chen H., Wang X., Heng J. (2009). Removal of aqueous Hg(II) by polyaniline: Sorption characteristics and mechanisms. Environ. Sci. Technol..

[48] Hernandez-Morales V. (2008). Synthesis and Characterization of the Gold Catalysts Supported on SBA–15 Modified with SH Groups. Master’s Dissertation.

[49] Boss C.B., Fredeen K.J. (1997). Concept, Instrumentation and Techniques in Inductively Coupled Plasma Optical Emission Spectrometry.

[50] Brunauer S., Emmett P.H., Teller E. (1938). Adsorption of gases in multimolecular layers. J. Am. Chem. Soc..

[51] Barrett E.P., Joyner L.G., Halenda P.P. (1951). The determination of pore volume and area distributions in porous substances. I. Computations from nitrogen isotherms. J. Am. Chem. Soc..

[52] Thommes M., Kaneko K., Neimark A.V., Olivier J.P., Rodriguez-Reinoso F., Rouquerol J., Sing K.S.W. (2015). Physisorption of gases, with special reference to the evaluation of surface area and pore size distribution (IUPAC Technical Report). Pure Appl. Chem..

[53] Nooney I.R., Kalyanaraman M., Kennedy G., Edward J. (2001). Heavy metal remediation using fuctionalized mesoporous silicas with controlled macrostructure. Langmuir.

[54] Acosta-Silva Y.J., Nava R., Hernández-Morales V., Macías-Sánchez S.A., Pawelec B. (2013). TiO_2_/DMS–1 disordered mesoporous silica materials: Structural characteristics and methylene blue photodegradation activity. Micropor. Mesopor. Mater..

[55] Boutros M., Onfroy T., Da Costa P. (2010). Mesostructured or alumina-mesostructured silica SBA–16 as potential support for NO_x_ reduction and ethanol oxidation. Catal Lett..

[56] Stevens W.J.J., Lebeau K., Mertens M., Van Tendeloo G., Cool P., Vansant E.F. (2006). Investigation of the morphology of the mesoporous SBA–16 and SBA–15 material. J. Phys. Chem. B.

[57] Mauraya M.R., Chandrakar A.K., Chand S. (2007). Oxovanadium(IV) and copper(II) complexes of 1,2-diaminocyclohexane based ligand encapsulated in zeolite Y for the catalytic oxidation of styrene, cyclohexene and cyclohexane. J. Mol. Catal. A Chem..

[58] Colthup B.N., Daly H.L., Wiberly E.S. (1990). Introduction to Infrared and Raman Spectroscopy.

[59] Jesenák K.L., Kuchta L., Hudec P., Fagnor V.S. (1999). Calcination of SiO_2_-Aerogel in Oxidizing Atmosphere. J. Therm. Anal. Cal..

[60] Kouznetsova T., Sauka J., Ivanets A. (2021). Template synthesis and gas adsorption properties of ordered mesoporous aluminosilicates. Appl. Nanosci. (Switzerland).

[61] Łuczkowski M., Stachura M., Schirf V., Demeler B., Hemmingsen L. (2008). Design of Thiolate Rich Metal Binding Sites within a Peptidic Framework. Inorg. Chem..

[62] Abdulghani A.J., Jasim H.H., Shebeeb Hassan A. (2013). Determination of Tetracycline in Pharmaceutical Preparation by Molecular and Atomic Absorption Spectrophotometry and High Performance Liquid Chromatography via Complex Formation with Au(III) and Hg(II) Ions in Solutions. Int. J. Anal. Chem..

[63] Nefedov V.I., Salyn Y.V., Zylberajch-Antoine C., Barraud A., Roulet H., Dufour G. (1999). XPS characterization of inserted mercury sulfide single layers in a Langmuir-Blodgett matrix. Appl. Surf. Sci..

[64] Castner D.G., Hinds K., Grainger D.W. (1996). X-ray photoelectron spectroscopy sulfur 2p study of organic thiol and disulfide binding interactions with gold surfaces. Langmuir.

[65] Humbert P. (1986). An XPS and UPS photoemission study of HgO. Solid State Commun..

[66] Mercier L., Pinnavaia T.J. (1997). Access in mesoporous materials: Advantages of a uniform pore structure in the design of a heavy metal ion adsorbent for environmental remediation. Adv. Mater..

[67] Antochshuk V., Olkhovyk O., Jaroniec M., Park I.S., Ryo R. (2003). Benzoylthiourea-modified mesoporous silica for mercury (II) removal. Langmuir.

[68] Green-Ruiz C. (2009). Effect of salinity and temperature on the adsorption of Hg(II) from aqueos solutions by Ca-montmorillonite. Environ. Tech..

[69] Hegazi H.A. (2013). Removal of heavy metals from wastewater using agricultural and industrial wastes as adsorbents. HBRC J..

[70] Jinjing L., Huazhen S., Hanna M., Zhongye W.N. (2011). Removal of Cu^2+^ from Aqueous Solution using Fly Ash. J. Miner. Mater. Character Eng..

[71] Ren X., Shi Y., Zheng H., Zhang H., Zuo Q. (2022). A novel covalent organic polymer with hierarchical pore structure for rapid and selective trace Hg(II) removal from drinking water. Sep. Purif. Technol..

[72] He H., Meng X., Yue Q., Yin W., Gao Y., Fang P., Shen L. (2021). Thiol-ene click chemistry synthesis of a novel magnetic mesporous silica/chitosan composite for selective Hg(II) capture and high catalytic activity of spent Hg(II) adsorbent. Chem. Eng. J..

